# Phylogenomics and gene association analysis support probiotic potential in a subset of Staphylococcus xylosus isolates

**DOI:** 10.1099/mgen.0.001708

**Published:** 2026-04-30

**Authors:** Kaylee Dawn Rich, Zhangbin Cai, Diego B. Nobrega, Jennifer Ronholm, Jeroen De Buck

**Affiliations:** 1 Faculty of Veterinary Medicine, Foothills Campus, University of Calgary, Calgary, AB, Canada; 2 Faculty of Agricultural and Environmental Sciences, Macdonald Campus, McGill University, Montréal, QC, Canada

**Keywords:** genomics, machine learning, mastitis, phylogenetics, probiotics, *Staphylococcus*

## Abstract

Mastitis is a major disease in dairy cattle, often caused by *Staphylococcus aureus* infection and increasingly complicated by antimicrobial resistance. As a result, bacterial strains able to effectively provide colonization resistance in the bovine mammary gland are being explored as potential probiotics to reduce reliance on antimicrobial treatments. Among these, some non-*S*. *aureus* staphylococci species have shown promise; however, their effects appear to be highly strain-dependent. In this study, a combination of machine learning approaches alongside standard genomic and phylogenetic analyses was used to compare and select putative probiotic *Staphylococcus xylosus* strains. The genomes of 82 S. *xylosus* and 16 closely related *Staphylococcus* isolates from milk samples obtained from the Canadian Bovine Mastitis Research Network were assembled and annotated. In addition to identifying orthologous gene families and reconstructing phylogenetic relationships, each genome was screened for virulence factors, antimicrobial resistance (AMR) genes and bacteriocins. Random forest modelling and association rule learning were then applied to identify combinations of genes associated with isolates from milk samples collected from quarters exhibiting low inflammation, assessed using somatic cell counts (SCC). This approach identified 63 genes that frequently co-occurred in isolates from low SCC samples (low SCC <25,000 cells ml^−1^) but were largely absent in those from high SCC samples (≥200,000 cells ml^−1^). These gene sets were used as biomarkers in conjunction with phylogenetic and clustering analyses to guide the selection of a subset of *S. xylosus* isolates with potential probiotic properties.

**Impact Statement**

Mastitis is a common and costly disease in dairy cows that affects milk production and animal health. *Staphylococcus aureus* is a major cause of bovine mastitis, but treatment success is often low, in part due to rising antimicrobial resistance, and new ways to prevent and manage infection are needed. One approach is the use of protective bacteria as probiotics. Non-*S. aureus* staphylococci (NAS) are common in the udder, but their effects vary widely between strains. While some NAS appear beneficial, others can still cause or exacerbate disease. Understanding the genetic basis of these differences is key to identifying strains suitable for probiotic development. We examined genomic variation among *Staphylococcus xylosus* and the closely related *Staphylococcus shinii* and *Staphylococcus pseudoxylosus* isolates to identify gene combinations associated with low inflammation in the udder, which may indicate probiotic potential. These findings could help detect protective NAS strains to use as probiotics that improve udder health without relying on antibiotics.

## Data Summary

Draft genome assemblies and raw reads can be found under National Center for Biotechnology Information (NCBI) BioProject PRJNA1336871, available at https://www.ncbi.nlm.nih.gov/bioproject/PRJNA1336871. Code and documentation for bioinformatic methods are available at https://github.com/kayleerich/biomARkers.

## Introduction

Mastitis is a costly disease in dairy cattle that is often caused by intramammary infection (IMI). It can present as either clinical mastitis, which shows visible signs such as swelling, redness and abnormal milk, or subclinical mastitis, which occurs without obvious symptoms but is typically detected through elevated somatic cell counts (SCC) [1,3]. This disease has significant impacts on animal health, milk production and farm economics. *Staphylococcus aureus* is a major cause of bovine mastitis, and though treating these infections with antimicrobials is common, bacteriological cure rates of IMI caused by *S. aureus* are often poor. Additionally, increasing antimicrobial resistance (AMR) has further challenged the success of therapies, and new treatment strategies are required [4,6]. Various approaches are being investigated, including the use of protective bacteria as a potential probiotic treatment [7,9].

Severity of IMI, including mastitis, is associated with microbiome composition in the udder, where some bacterial species appear to be protective against mastitis pathogens [10,13]. Among these are the frequently isolated non-*S. aureus* staphylococci (NAS). However, the relationships between NAS, other bacterial species and IMI severity are difficult to evaluate [2,10, 14]. The role of NAS in bovine udder health is complex and nuanced. While traditionally considered minor IMI pathogens and frequently implicated as IMI agents [15,17], some NAS strains may confer protective effects by inhibiting other mastitis-causing pathogens [18,19]. Recent studies have illustrated a strain-specific nature of NAS interactions with mastitis pathogens. For instance, Persson Waller *et al.* [20] found that certain *Staphylococcus chromogenes* genotypes were associated with more severe IMI than other *S. chromogenes* genotypes, whereas Jung *et al.* [12] found a negative correlation between NAS occurrence with *Escherichia coli* clinical mastitis, supporting the notion that some NAS may contribute positively to udder health. These accounts emphasize the need to distinguish between potentially virulent and protective NAS. They also support the increasingly accepted view that the effect of NAS on udder health is highly strain- or genotype-dependent [10].

Identifying beneficial NAS strains requires a deeper understanding of the mechanisms by which they interact with pathogenic species. Genes from protective strains could serve as biomarkers to predict a strain’s probiotic potential or its risk of contributing to severe IMI. Among the mechanisms of antagonism, several have been described, including quorum quenching, antimicrobial production and biofilm inhibition. Quorum quenching is the disruption of bacterial communication systems that regulate gene expression based on population density. By interfering with these signalling pathways, antagonistic bacteria have been shown to attenuate the virulence of other bacteria [21]. NAS are capable of inhibiting the *S. aureus* quorum-sensing system, which is responsible for the expression of a range of virulence factors by interfering with its accessory gene regulator (*agr*) [22,23]. By producing analogues of the *S. aureus* autoinducing peptides (AIPs) that mediate *agr* signalling, NAS suppress *S. aureus* quorum sensing and pathogenicity. The presence of AIP analogues in NAS genomes could be a promising lead in the search for probiotics. However, since these peptides target specific strains, reliance on AIP-producing NAS as a prophylactic may lead to the selection of AIP-resistant *S. aureus* strains [23].

Another antagonistic mechanism employed by NAS is the production of bacteriocins. These are antimicrobial peptides produced by a wide range of bacteria and have garnered attention for their potential in novel therapeutic development. NAS-derived bacteriocins have been shown to inhibit *S. aureus* [24], and bacteriocin-producing NAS have been proposed as potential probiotics [24,26]. However, the relationship between bacteriocin gene content and inhibitory activity is not straightforward. For instance, two strains of *Staphylococcus simulans* with differing bacteriocin gene profiles were found to have similar inhibitory effects on colonization of the mammary gland by *S. aureus* [24,27], indicating that other factors may contribute to their antagonistic activity *in vivo*. Recent studies also suggest that biofilm inhibition may play a more critical role in the prophylactic potential of certain NAS strains [28]. Though the underlying genes involved are unknown, this effect appears to be independent of bacteriocin production [29]. These findings indicate that while bacteriocins are a promising avenue for drug development, they are unlikely to be the sole determinant of probiotic efficacy.

The mechanisms underlying the effect that NAS have on IMI are complex, and a complete understanding of the genes involved remains elusive. While some NAS strains inhibit mastitis-causing pathogens, they may still possess pathogenic potential themselves. Additionally, interactions with the host immune response must also be considered when evaluating probiotic suitability [30]. For this reason, focusing solely on genes with known functions may overlook subtle but important genetic patterns that contribute to protective effects. In this study, we focused on the NAS species *Staphylococcus xylosus* as a prospective probiotic because it is one of the most common NAS species isolated from bovine milk samples, it has been associated with lower milk SCC than the other commonly-isolated NAS species *S. chromogenes* and *S. simulans* and it is not associated with persistent IMI [31,32]. Furthermore, though the reported effect that *S. xylosus* has on udder health varies, some strains have been shown to interfere with *S. aureus* quorum sensing and biofilm formation [13,33,35]. To identify strains with probiotic potential, we characterized genomic and phylogenetic diversity within *S. xylosus* and two species formerly considered conspecific with *S. xylosus: Staphylococcus shinii* and *Staphylococcus pseudoxylosus*. Because these taxa are highly similar (ANI >90%) but differ in SCC phenotypes, we included them to improve the stratification of isolates and enhance our ability to distinguish genes associated with high versus low SCC. We used SCC to categorize isolates according to host inflammatory response. Using random forest modelling and association rules learning to identify groups of genes associated with low inflammation, we aimed to identify genetic markers that could differentiate protective from potentially harmful *S. xylosus* strains.

## Methods

### Genome extraction, isolation and sequencing

The 98 *Staphylococcus* isolates used in this study were sourced from the Mastitis Pathogen Culture Collection located at the Faculté de médecine vétérinaire à Saint-Hyacinthe in Québec, and data and samples were originally collected during a study conducted by the National Cohort of Dairy Farms of the Canadian Bovine Mastitis Research Network [36]. Isolates were divided into two groups based on sample SCC of the corresponding milk samples: SCC_low_ <25,000 cells ml^−1^ or SCC_high_ ≥200,000 cells ml^−1^. Genomic DNA was extracted from isolates using the DNeasy UltraClean Microbial Kit (QIAGEN, Germany) according to the manufacturer’s instructions. Libraries were prepared with the Nextera DNA Flex kit (Illumina, USA) and sequenced on an Illumina MiSeq to generate 2×300 bp paired-end reads.

### Whole-genome assembly and annotation

Snakemake v7.32.4 [37] was used to automate the assembly and annotation process for all isolates. The raw sequence reads were screened for low quality and adapter sequences and then trimmed using Fastp v0.24.0 [38,39]. Quality of the trimmed sequences was assessed with FastQC v0.12.1 [40] then *de novo* genomes were assembled using SPAdes v4.0.0 [41] implemented by Unicycler v0.5.1 [42] with default parameters. Genome assembly quality and completeness were assessed using Quast v5.3.0 [43,44] and BUSCO v5.8.0 [45] (parameters: -m geno -l bacillales_odb10), and contigs shorter than 500 bp were removed using seqkit v2.5.1. Annotation of protein-coding genes, rRNAs and tRNAs in the assembled genomes was done using Pyrodigal v3.6.3 [46], Infernal v1.1.5 [47] and tRNAscan v2.0.12 [48] implemented via Bakta v1.10.1 [49] (additional parameters: --genus Staphylococcus --species xylosus --gram +, full database). The species of each isolate was assessed by calculating average nucleotide identity (ANI) between each genome and the reference genomes for *S. xylosus*, *S. shinii* and *S. pseudoxylosus* (NCBI accessions: GCF_000709415.1, GCA_017583065.1, GCF_044794325.1), where two genomes were considered from the same species if ANI >95%.

Plasmid sequences were identified and extracted from the genome assembly of each isolate using MOB-suite v3.1.9 [50]. The identified plasmid sequences were manually examined, and those that were annotated solely with non-plasmid genes (e.g. rRNA genes, MOB closest-neighbour match to NCBI accession CP042023, CP042061 or CP042065) were removed. Differences in the distributions of the number of plasmids per isolate between high and low SCC isolates were assessed using the exact two-sample Kolmogorov−Smirnov test.

Virulence factors, bacteriocins and AMR genes were identified using a manually curated database that included sequences from VRprofile, BAGEL4 [51] and CARD [52] databases, based on predicted orthology (see the next section for orthology prediction methods).

### Pan-genome and phylogenetic analysis

Orthologous gene families (orthogroups) were determined via Orthofinder v3.0.1 [53,54] using the annotated protein-coding sequences obtained from Bakta. Orthogroups were then used to characterize the pan-genome of *S. xylosus*, represented by the core genome (hard core: shared by ≥99% of isolates, soft core: 99%>isolates≥95%), accessory genome (95%>isolates≥15%) and cloud genome (shared by <15% of isolates). Pan-genome analyses were assessed in R using the pagoo v0.3.18 [55] and micropan v2.1 [56] packages.

To assess diversity and evolutionary relationships among the *S. xylosus*, *S. shinii* and *S. pseudoxylosus* isolates, phylogenetic trees were constructed from single-copy protein-coding genes present in ≥95% of isolates. The *S. xylosus* reference genome (accession GCF_000709415.1) was included for the phylogenetic context of the isolates’ placement within the tree. The coding sequences of annotated protein-coding genes were clustered using CD-HIT v4.8.1 [57,58] at ≥90% sequence identity over ≥80% sequence length (parameters: -c 0.90 s 0.80 n 5). Sequences in clusters with only one representative from ≥95% of isolates were used to generate a database of marker proteins for phylogenetic tree construction with PhyloPhlAn v3.1.1 [59] (non-default parameters: --db_aa diamond --min_num_entries 85 --diversity low –trim greedy --remove_fragmentary_entries --fragmentary_threshold 0.67). Visual comparisons to the gene-tree-derived isolate tree generated by Orthofinder were done to assess if there were notable discrepancies between the two. Trees were visualized in R using the ggtree package v3.16.0 [60,61].

### Isolate similarity and orthogroup correlations

Multidimensional scaling (MDS) for all isolates was done using accessory genome orthogroups (in <95% of isolates), as well as all virulence factor, AMR and bacteriocin orthogroups. MDS distances and *k*-means clusters from those distances were calculated using the cmdscale and *k-*means functions from the stats package v4.3.2 in R. The *k*-means clusters for the virulence factor, AMR and bacteriocin MDS distances were coloured based on similarity to the accessory genome *k*-means clusters. Similarity was determined by considering the number of shared isolates between clusters, guided by calculating the Rand Index for cluster similarity using the rand_score function from the scikit-learn module v1.4.2 in Python, and plots were visualized using ggplot2 v3.5.2 in R.

Pairwise correlation for all virulence factors, AMR and bacteriocin orthogroups was determined by calculating the phi coefficient with a confidence level of 0.95 for orthogroup presence in SCC_high_ and SCC_low_ isolates using the cor function of the stats package v4.3.2 in R. The plot was visualized in R using corrplot v0.95.

### Biomarker identification using machine learning algorithms

A random forest (RF) classifier model was trained using the presence or absence of all orthogroups to identify important predictors for SCC level. The model was implemented in Python 3 using the RandomForestClassifier from the scikit-learn module v1.4.2 [62]. To improve computational efficiency, two filtering steps were applied: (i) orthogroups shared by more than 95% and fewer than 5% of isolates were removed, and (ii) one representative was chosen for groups of orthogroups that were present/absent in all of the same isolates. To mitigate the risk of overfitting the model to this dataset, the data were split, and 80% of the isolates were used to train the model, while the remaining 20% of isolates were used to test the resulting model for precision and accuracy.

To differentiate orthogroup presence that was predictive for either low or high SCC (SCC_low_ or SCC_high_), a random forest clustering method was implemented using the FGClustering module v1.1.1 [63] in Python. The resulting RF groups were analysed to determine isolates grouped based on SCC level. These were labelled as RF_high_ and RF_low_ for the group with the highest number of isolates from high and low SCC samples, respectively. Each isolate was designated as SCC_low_/RF_high_, SCC_low_/RF_low_, SCC_high_/RF_low_, or SCC_high_/RF_high_ to indicate its SCC status and RF group. Isolates where SCC and RF group did not match (SCC_low_/RF_high_ and SCC_high_/RF_low_) were considered more likely to have had their SCC status influenced by host physiological state and environmental exposures (such as immune competence, stress responses, management conditions, co-infection or microbiome composition) rather than isolate genomic features and were not used in the next steps.

Associations between important predictive orthogroups (*P*-value<0.001) for each group were identified using the frequent pattern growth and association rules learning algorithms (fpgrowth and association_rules) from the mlxtend Python module v0.23.4 [64]. Rules were filtered by *lift* (a measure of how often orthogroups in the set are present in the same isolate than would be expected if they were statistically independent) and *confidence* (the probability of seeing that orthogroup in an isolate given that the other orthogroup is also present) scores, where *lift* ≥1.3 and *confidence* ≥0.8. Rules for the RF_low_ group were examined, and those containing an orthogroup also appearing in a rule from the RF_high_ group were removed. The remaining rules with *lift* >1.5 were used to identify unique combinations of five orthogroups.

Data management was primarily done in Python using pandas v2.3.1 and numpy v1.24.3. Bar graphs and pie charts were generated in Python using matplotlib v3.7.1, and the networks were visualized using the gravis v0.1.0 module [65].

### Data Availability

Draft genome assemblies and raw reads were deposited under BioProject PRJNA1336871 [https://www.ncbi.nlm.nih.gov/bioproject/PRJNA1336871]. BioSample accessions for each isolate are provided in Table S1 (available in the online Supplementary Material). Code and documentation are available at https://github.com/kayleerich/biomARkers.

## Results

### Pangenome analysis

After genome assembly and annotation, we assessed the completeness of each assembly using BUSCO and calculated an average completeness score of 98.7% (99.3–100%) (Table S1). Across the 98 isolates, we found an average of 2679 protein-coding genes, 58 tRNA genes and 20 rRNA genes per isolate. We confirmed the species of each isolate via ANI and found that the *S. xylosus* isolates had an average ANI of 98.58%, 93.46% and 91.22% to the *S. xylosus*, *S. pseudoxylosus* and *S. shinii* reference genomes, respectively. ANI between the *S. xylosus* reference genome and the *S. pseudoxylosus* or *S. shinii* isolates was 93.47% and 91.20%, respectively.

**Fig. 1.**
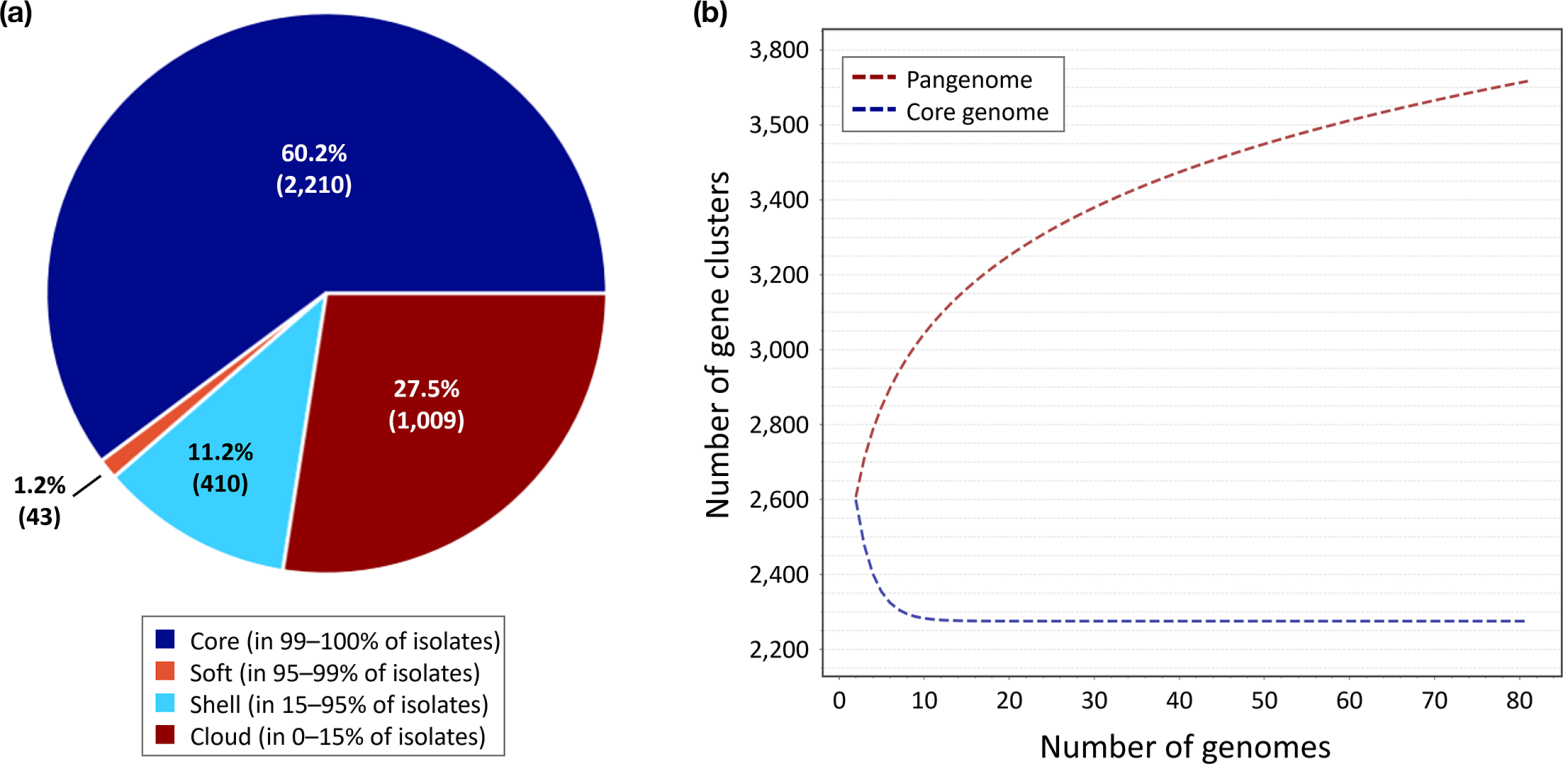
Pangenome of 82 *S*. *xylosus* isolates. (**a**) Pangenome composition of core (dark blue), soft-core (orange), shell (light blue) and cloud (red) orthogroups. (**b**) Rarefaction curve for 77 permutations of 82 isolates depicting the change in the total number of orthogroups in the pangenome (red) and the core genome (blue) with the addition of each *S. xylosus* genome.

We identified 3,672 protein-coding orthogroups in the pangenome of 82 *S*. *xylosus* isolates (Table S2). From these, we identified genes belonging to the core (present in ≥95% of isolates, *n*≥78) and accessory genomes (present in <95% of isolates, 1≤*n*>78), where 2,253 (61.4%) orthogroups were part of the core genome and 1,419 (38.6%) were part of the accessory genome. Of the core genome, we found that only 43 (1.2%) orthogroups were present in 95–99%, designated the soft-core genome (78≤*n*>81). We further divided the accessory genome into the shell (present in 15–95% of isolates, 12≤*n*>78) and cloud (present in 0–15% of isolates, 1≤*n*>12) genomes, which contained 410 (11.2%) and 1,009 (27.5%) orthogroups, respectively (Fig. 1a). Of the latter, 58 were unique, i.e. found in only one isolate. When genomes were added one at a time, we found that the number of core orthogroups decreased until the addition of new genomes had no further effect. The total number of orthogroups in the pangenome continued to increase with each inclusion (Fig. 1b), indicating an open pangenome (Heap’s law *α*=0.95).

### Phylogenetic analysis and clustering

**Fig. 2.**
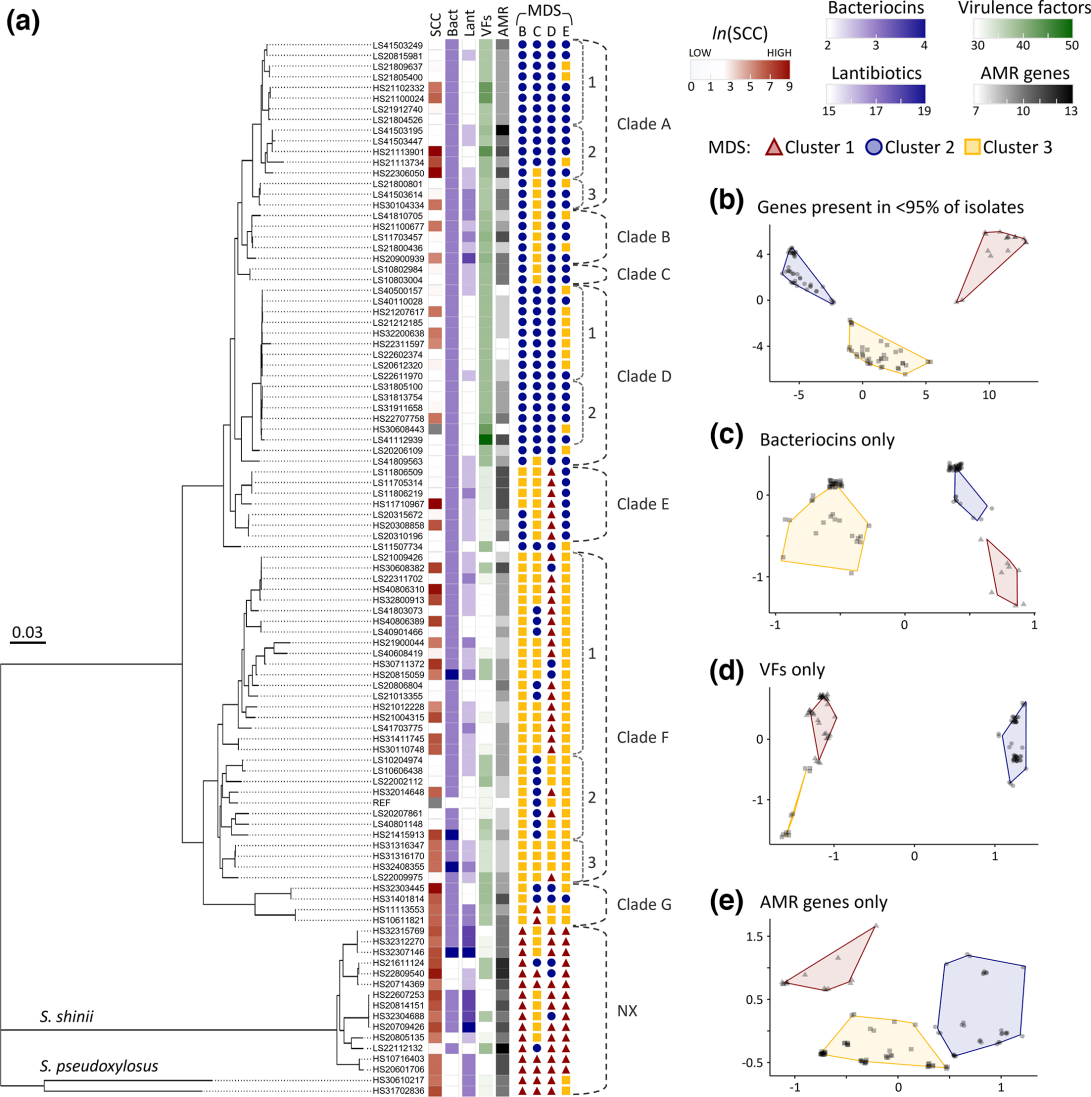
Phylogeny and MDS clustering of 98 *Staphylococcus* isolates. (**a**) Phylogenetic tree split into seven distinct *S. xylosus* clades, labelled Clades A through G. *S. shinii* and *S. pseudoxylosus* clade branches are labelled with species name and collectively referred to as NX. Phylogeny was generated with Phylophlan using 1,816 markers comprised of 43,839 aligned positions from proteins present in ≥95% of isolates. The heatmap displays the SCC (where darker red indicates higher SCC) for each isolate, and the number of bacteriocins and lantibiotics (purple), virulence factors (green) and AMR (black) orthogroups present in its genome. Symbols correspond to *k*-means clusters of MDS distances in (b–d) (red/triangles, cluster 1; blue/circles, cluster 2; yellow/squares, cluster 3) to visualize whether the different gene categories (accessory genes, bacteriocin genes, virulence genes or AMR genes) follow similar patterns between and within phylogenetic clades and clustering in the MDS plots. MDS distances were calculated as a measure of similarity between isolates using (b) orthogroups present in at least 5% of isolates and less than 95% of isolates, (**c**) bacteriocin orthogroups only, (**d**) virulence factor orthogroups only and (e) AMR gene orthogroups only.

Using the orthogroups present in more than 95% of the isolates (*n*=2,279), we constructed a phylogenetic tree with PhyloPhlAn (Fig. 2a). From this phylogeny, we identified seven distinct clades, which we labelled A through G. These formed two separate lineages; one comprised of Clades A through E and the other of Clades F and G. We also found that the *S. shinii* and *S. pseudoxylosus* isolates each formed their own outgroup clades, which we collectively refer to as NX (non-*xylosus*) for the sake of clarity. We then calculated MDS distances for all isolates using orthogroups present in <95% of the isolates and found that, except for Clade E, isolates in a phylogenetic clade clustered together (Fig. 2b).

We observed some clear distinctions between clades. All but one of the NX isolates were SCC_high_, and these isolates formed a clear group (*k*-means cluster 1) using genes present in <95% of all isolates (Fig. 2b). Clade F contained both SCC_low_ and SCC_high_ isolates, which were not segregated by subclade. They also did not cluster with Clades A–D after MDS analysis. Instead, we found that Clade F clustered more closely with Clade G and with some of the isolates from Clade E (*k*-means cluster 2). The remaining Clade E isolates clustered with Clades A–D (*k*-means cluster 3), which were primarily SCC_low_.

When we used only virulence factor (VF), AMR or bacteriocin orthogroups to calculate the MDS distances between isolates (Fig. 2c–e), the *k*-means clusters did not correspond to the phylogeny as strictly. We also noticed that the *k*-means clusters were not consistent with the number of orthogroups present in each isolate. Though the NX isolates and Clade E had the most AMR genes (10–13 and 9–11, respectively), the isolates from these clades clustered separately, indicating different AMR gene profiles for these groups. Clade A isolates also had more AMR genes than the remaining clades (8–13), and the majority of these isolates clustered with Clade E. Isolates in Clades A, B, C and D were primarily SCC_low_ and had between 38 and 49 virulence factor orthogroups each. All of the isolates in these clades clustered together, indicating similar virulence factor profiles. This was different from our observations of NX and Clade E isolates, which clustered together and had fewer virulence factors – between 32–39 and 32–34, respectively. However, the NX clades are characterized by SCC_high_ isolates, and all but one of the isolates from Clade E were SCC_low_.

For all previously discussed MDS distance clustering, the NX isolates largely clustered together. Interestingly, we did not observe this trend when we calculated the MDS distances using only the presence of bacteriocin genes (Fig. 2c). The NX isolates had the greatest variation in the number of encoded bacteriocin genes, and those in the same *k*-means cluster were not the most closely related isolates. Lantibiotic presence in particular varied greatly in NX isolates, ranging from 15 to 19 lantibiotic genes encoded. In comparison, isolates from the other clades encoded a maximum of 17 lantibiotics, and within-clade variation was only 3 for Clade B, 2 for Clade A, 0 for Clade C and 1 for all other clades.

### Associations between virulence factors, AMR and bacteriocin orthogroups

We did not find a significant correlation between SCC level and number of virulence factors or bacteriocins. We did, however, find a positive correlation between SCC and number of AMR genes (*R*^2^=0.28, *P*=0.0059) and number of AMR genes and lantibiotics (*R*^2^=0.31, *P*=0.0019) as well as a negative correlation between number of VFs and lantibiotics (*R*^2^=−0.41, *P*=4.44e−05).

**Fig. 3.**
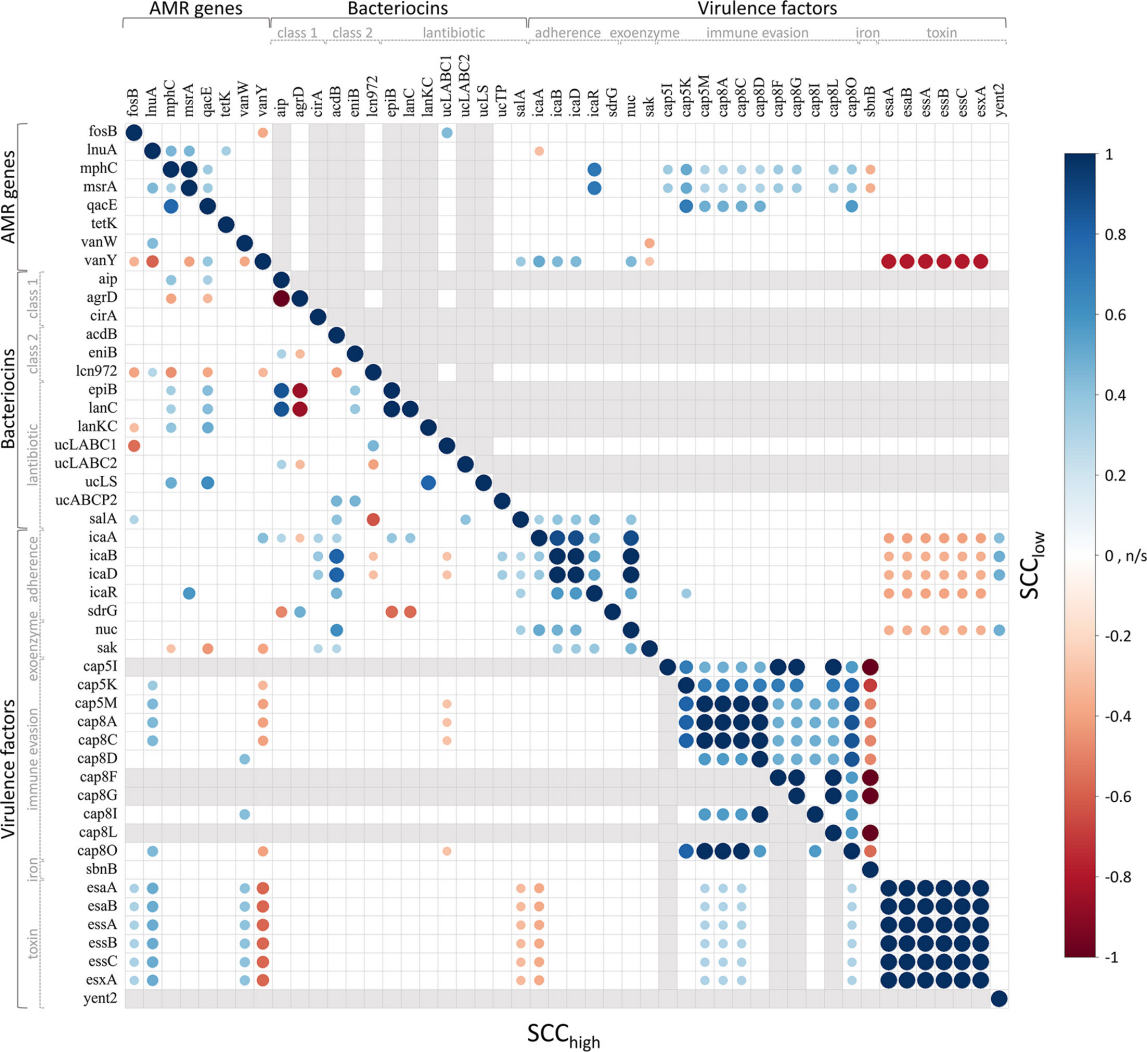
Significant pairwise associations between AMR genes, bacteriocins and virulence factors for SCC_high_ isolates (bottom left matrix) vs. SCC_low_ isolates (top right matrix). Associations (*P*>0.05) are represented by phi correlations for the presence of orthogroups, and positive association is represented in blue and negative in red. The column or row is greyed out if the orthogroup was not found in that group of isolates (i.e. SCC_high_ or SCC_low_). Orthogroup IDs associated with gene symbols can be found in Table S3.

When we did pairwise comparisons of the presence and associations between bacteriocin-encoding genes, we found that there were some bacteriocins not encoded by any SCC_low_ isolates, and no significant associations between the bacteriocins were present (Fig. 3). In contrast, there were multiple associations between bacteriocins in SCC_high_ isolates. These included positive associations between autoinducing peptide IV (*aip*), enterocin X-*β* (*eniB*), lantibiotic synthetase component C (*lanC*) and lantibiotic dehydratase/lantibiotic epidermin biosynthesis protein (*epiB*), which were all negatively associated with cyclic lactone autoinducer peptide (*agrD*).

We found that associations between capsule orthogroups such as *cap8A*, *cap8C*, *cap8D* and *cap5M* were prevalent in both SCC_low_ and SCC_high_ isolates. However, other capsule genes (*cap8F*, *cap8G*, *cap8L* and *cap5I*) were not found in isolates from SCC_high_ samples. We also found that another capsule gene, *cap5K*, was positively associated with the quaternary ammonium compound resistance gene *qacE* in SCC_low_ isolates but had a weak negative association in SCC_high_ isolates.

In both SCC_low_ and SCC_high_ isolates, we found that type VII secretion system (T7SS) genes (*esaA*, *esxA*, *essA*, *esaB* and *essC*) were negatively associated with the vancomycin resistance gene *vanY*. These T7SS genes were positively associated with other AMR genes, including another vancomycin resistance gene (*vanW*), a thiol transferase conferring fosfomycin resistance (*fosB*) and lincosamide nucleotidyltransferase (*lnuA*) in SCC_high_ isolates with no significant association in SCC_low_ isolates. *lnuA* was also the only gene significantly positively associated with the tetracycline resistance gene *tetK* and only in SCC_low_ isolates.

### Plasmids

**Fig. 4.**
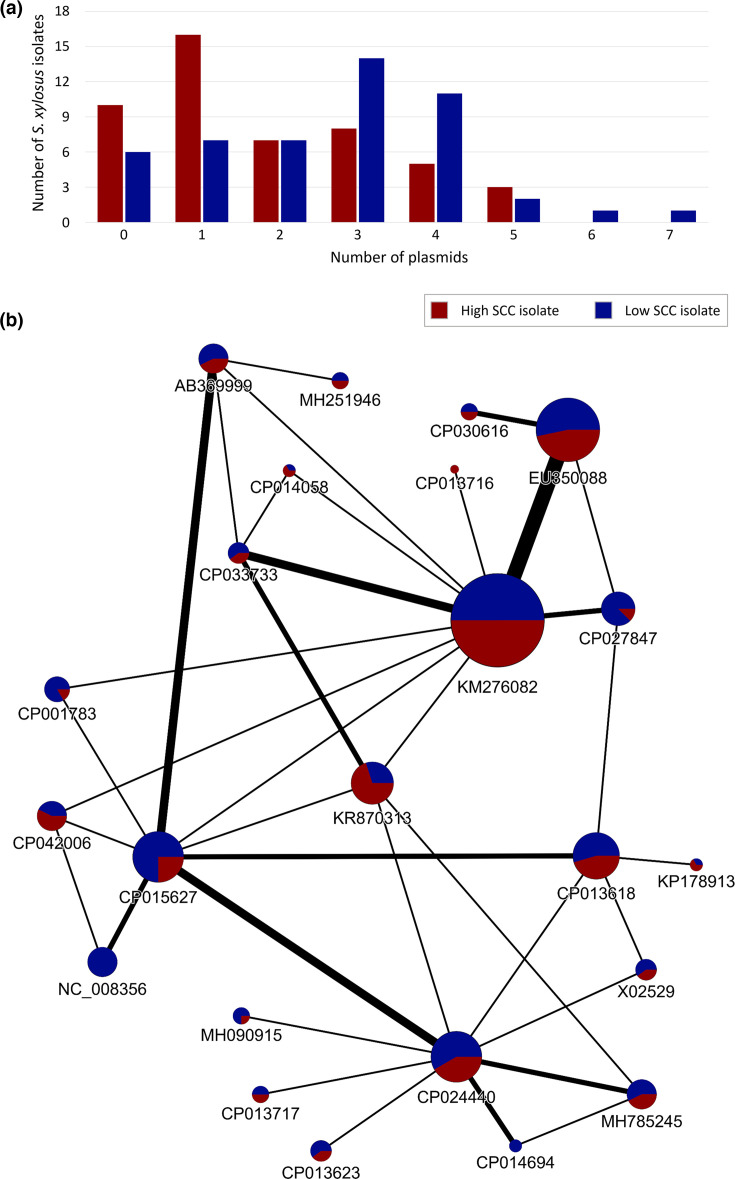
(a) Number of plasmids per isolate coloured by either SCC_high_ (red) or SCC_low_ (blue). (**b**) Network of plasmids found in the same isolate. Each node represents a plasmid, with size indicating the number of isolates carrying that plasmid and colours representing the proportion of SCC_high_ (red) or SCC_low_ (blue) isolates. Edges link co-occurring plasmids, with thickness representing the number of isolates in which that pair of plasmids was found. The network has been filtered to remove edges representing a single link and plasmids only found in a single isolate.

When we examined the association between *lnuA* and *tetK* further, we found that both genes were commonly encoded on plasmids. Though they were never encoded on the same plasmid, we found that 15 isolates (12 SCC_low_, 3 SCC_high_) carried a plasmid for each gene. Furthermore, we found that there were no orthogroups solely encoded on plasmids and that SCC_low_ isolates carried more plasmids than SCC_high_ isolates (exact two-sample Kolmogorov–Smirnov test, *P*=0.02597) (Fig. 4a). We also found that the median number of plasmids per isolate was 2 and 16 isolates had no identified plasmids. Though there were plasmids that we found only in SCC_low_ or SCC_high_ isolates, such as NC_008356, which we found in 7 SCC_low_ isolates, there was no single plasmid found in every SCC_low_ or SCC_high_ isolate. We also observed that some plasmids were never carried together by the same isolate, and some plasmids were only carried by *S. xylosus* or NX isolates. For example, we found the plasmids EU350088 and CP024440 in 15 and 12 isolates, respectively, but never in the same isolate (Fig. 4b). Of the two, EU350088 was only found in *S. xylosus* isolates, but CP024440 was found in both *S. xylosus* and NX isolates.

### Random forest modelling and clustering

There were no orthogroups that were found in all SCC_low_ isolates and not in any SCC_high_ isolates. Due to the limitations of using SCC as a proxy for NAS-induced IMI, we sought to reduce single-sample noise from external factors by enriching for isolates carrying genes consistently associated with low SCC. To this end, we trained an RF classifier followed by RF-based clustering to identify genes whose presence was an important predictor for SCC_low_ and grouped isolates based on similarity in decision paths within the model. The overall accuracy of our RF model was 0.85, with 0.89 for target (i.e. SCC_low_) precision and 0.90 for target recall. We identified two groups within the RF model: the first consisted of 34 SCC_high_ isolates and 6 SCC_low_ isolates and the second consisted of 15 SCC_high_ isolates and 43 SCC_low_ isolates. Since the first group contained the majority of SCC_high_ isolates and the second group most of the SCC_low_ isolates, we refer to these as RF_high_ and RF_low_, respectively. All the NX isolates and ~30% of the *S. xylosus* isolates were included in RF_high_, indicating that the primary influence on RF clustering was not phylogenetic distance.

**Fig. 5.**
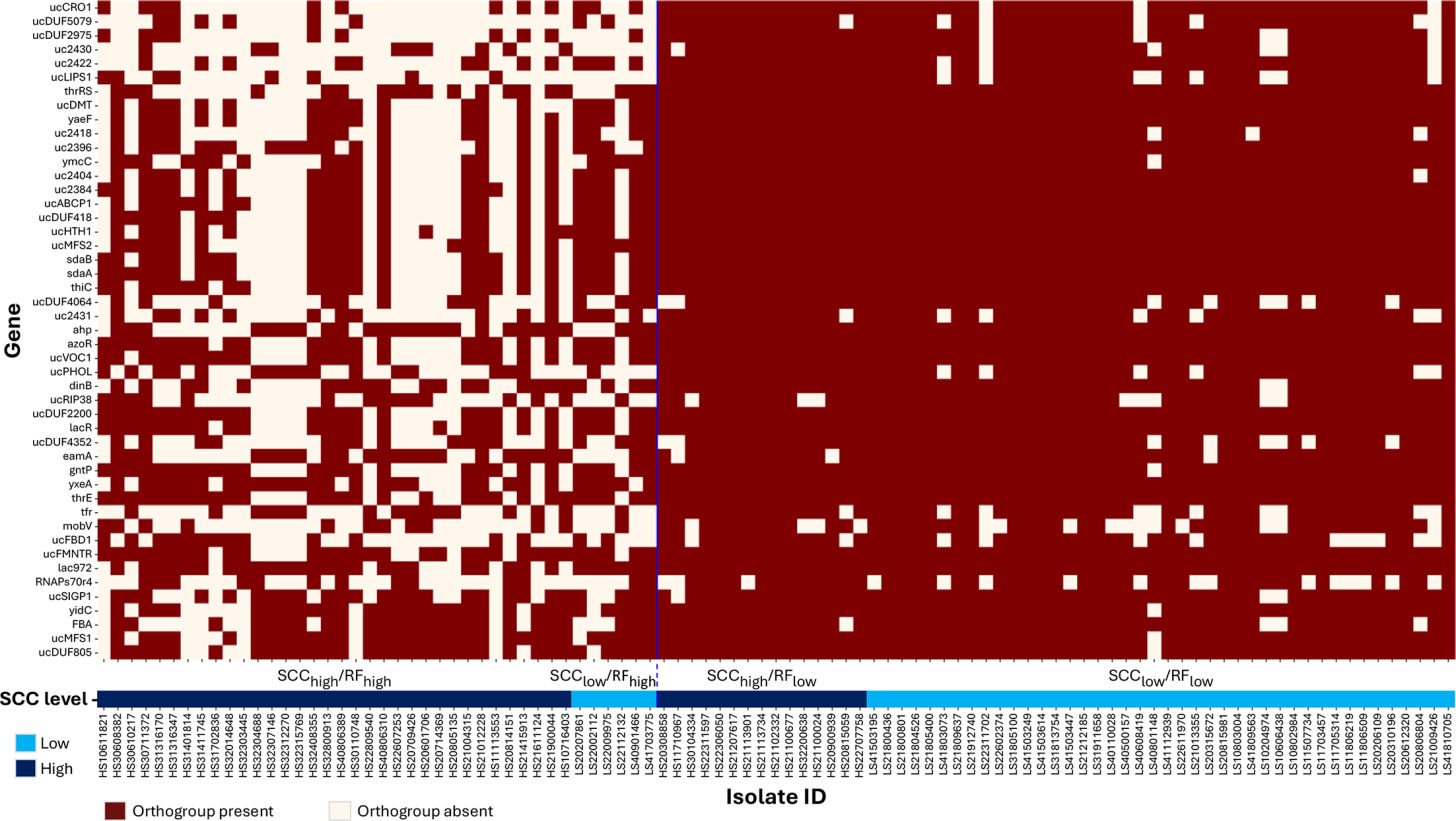
Heatmap for the presence (red) or absence (white) of orthogroups that were important for accurately predicting SCC level by the random forest model. The *S. xylosus* isolates fell into two subgroups based on similar decision pathing in the RF model and the SCC level is indicated in blue (SCC_high_ by dark blue and SCC_low_ by light blue). The orthogroups shown are those whose presence was a significant predictor for the RF_low_ group but not significant for the RF_high_ group (*P*<0.001).

We selected orthogroups that were important predictors for RF_low_ (*P*<0.001) as candidate biomarkers for SCC_low_ (Fig. 5). Some notable orthogroups included a TetR/AcrR family transcriptional regulator (*tfr*, OG0002443), lactococcin 972 (*lcn972*, OG0002332), alkylhydroperoxidase (*ahp*, OG0002375) and an FMN-dependent NADH:quinone oxidoreductase (*azoR*, OG0002367).

### Frequent patterns and association rules

To find orthogroups that are commonly found in the same isolates (co-occurring biomarkers), we implemented two algorithms: frequent pattern growth (FP) to calculate the frequency of all combinations of orthogroups for each group and association rules machine learning (ARL) to find statistically significant combinations. These two algorithms use a significant amount of computing power; the amount of time and memory required for FP and ARL calculation increases exponentially with the number of items in the dataset. This has posed a substantial obstacle in applying these algorithms to large genomic datasets. Therefore, to reduce the complexity of the input dataset, we chose to only use orthogroups that were identified as important predictors (*P*<0.001) by the clustered RF model: 80 for RF_high_ and 149 for RF_low_.

**Fig. 6.**
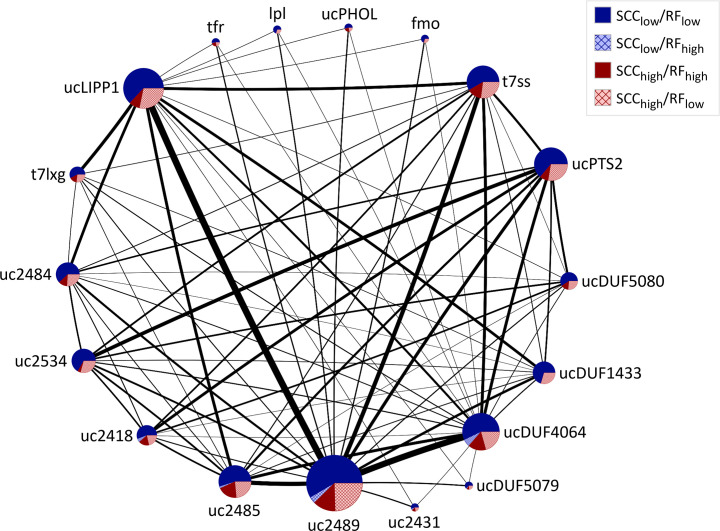
Network of orthogroups from the set of potential biomarker groups. Nodes represent single orthogroups with colours representing SCC_low_/RF_low_ (dark blue), SCC_high_/RF_high_ (dark red), SCC_low_/RF_high_ (hatched blue) or SCC_high_/RF_low_ (hatched red) isolates; edges connect orthogroups that appear together in the final set of association rules. Node size is indicative of the relative number of rules containing that orthogroup, and edge thickness indicates the relative number of times those two orthogroups appear in the same rule. Network has been filtered for readability to only include edges and nodes for orthogroups that appeared with more than one of the other 62 orthogroups in ≥200 rules. Orthogroup IDs associated with gene symbols can be found in Table S3.

After calculating frequencies and association rules (maximum number of genes per rule=5) for each RF group, we filtered by *lift,* where *lift* >1 indicates a positive correlation for orthogroup presence in the same isolate, and removed any rules for RF_low_ containing an orthogroup that also appeared in a rule for RF_high_, since these are not informative for differentiating between SCC_low_ and SCC_high_ isolates. This reduced the number of association rules from 170,369,670 to 161,690, and we identified 6,455 potential biomarker sets created from 63 orthogroups when we filtered for rules with unique combinations of 5 genes and *lift* >1.5. From the final set of rules, we identified the orthogroups most frequently found in association rules together (Fig. 6, Table S3). Among these, we found multiple notable associated orthogroups, including the T7SS proteins (*esaA*, *esxA*, *essA*, *esaB*, *essB* and *essC*; collectively referred to as T7SS), *tfr*, lysophospholipase (*lpl*) and T7SS effector LXG polymorphic toxin (*t7lxg*).

We further investigated which isolates encoded different combinations of the 63 orthogroups, as isolates encoding these gene combinations may be suitable candidates for use as probiotics. As expected, we found that combinations of these orthogroups were encoded by more SCC_low_ than SCC_high_ isolates. For example, while individual orthogroups containing the genes *t7lxg*, *lac972*, *tfr*, *azoR* and T7SS were common in both SCC groups (in 41–80% of SCC_high_ isolates and 59–100% of SCC_low_), the co-occurrence of all five was found in just 24% of SCC_high_ isolates but 56% of SCC_low_ isolates. We found that all of the SCC_high_ isolates encoding this combination of orthogroups were from the RF_low_ group, as depicted in Fig. 5. Another gene combination of *tfr*, signal transduction protein TRAP and 2-hydroxy-3-oxopropionate reductase *garR* was encoded by 17 SCC_high_ isolates and 33 SCC_low_ isolates. Three of the SCC_high_ isolates were SCC_high_/RF_high,_ though the remaining 14 SCC_high_ isolates and all the SCC_low_ isolates belonged to RF_low_. When we considered isolates that encoded T7SS genes as well, the number of SCC_high_ isolates dropped to 12, all of which belonged to RF_low_. Similarly, 33 SCC_low_ isolates and 14 SCC_high_ isolates (69% of SCC_low_ isolates compared to only 29% of SCC_high_ isolates) encoded the combination of *fmo*, *lac972* and damage-inducible protein DinB (*dinB*). When we examined these isolates further, we again found that all of them belonged to RF_low_. Interestingly, 97% of all isolates carry the gene for cyclic lactone autoinducer peptide (*agrD*). However, when we looked at the presence of *agrD* with *t7lxg*, we found that both were present in 77% of the RF_low_ group, but only 20% of RF_high_ isolates carried both.

Though the number of isolates, particularly NX isolates, included in this study is insufficient to determine whether a gene is *S. xylosus*-specific, we investigated whether any of the 63 genes were found in all *S. xylosus* isolates but not in any NX isolates. We found that only one (*thiC*) was carried by every *S. xylosus* isolate and no NX isolates, and two uncharacterized genes (*uc2384* and *ucABCP1*) were found in isolates from every *S. xylosus* phylogenetic clade but not found in any NX isolates.

### Probiotic evaluation

To identify the specific isolates with probiotic potential, we examined the phylogenetic relationships, results from our MDS analyses, random forest isolate clustering and the presence of gene combinations identified through association rules learning. We also considered isolates with fewer AMR genes to be preferential due to the positive correlation we found between the number of AMR genes and SCC, but also because a probiotic carrying AMR genes has the potential to transfer those genes to more pathogenic bacteria. Taking these factors into account, we excluded all NX from consideration since there were vastly more SCC_high_ NX isolates than SCC_low_ isolates and all NX isolates belonged to RF_high_. Similarly, Clades F and G contained more SCC_high_ isolates than SCC_low_ isolates, and no Clade F subclades contained isolates only belonging to RF_low_, which gave us less confidence in selecting any of these isolates as a potential probiotic. All of the isolates in the remaining clades belonged to RF_low_. From the remaining clades, we considered similarity in the presence of VFs, bacteriocins or AMR genes to the NX clades, Clade F or Clade G as a possible exclusion factor (i.e. isolates that clustered with Clades F, G or NX in the MDS analyses). Consequently, we excluded Clade E because these isolates clustered with the presence of the NX virulence factor in the MDS analyses.

After these exclusions, we looked at the presence of the aforementioned genes and gene combinations in the isolates of each clade. We found that the T7SS protein-encoding genes were present in all Clade A, B, C and D isolates but missing from all isolates in Clades F2 and E, as well as all NX isolates. Similarly, both *fmo* and *lpl* were missing from isolates in Clades F, G and NX but present in all others. Furthermore, none of the NX, Clade F or G isolates and less than half of the isolates in Clade E contained any of the gene combinations detailed in Table 1. Though it is impractical to compare and document all combinations – we have provided a parsing tool to investigate gene combinations in the supplemental materials – isolates from Clades A1, B and D1 consistently encoded all genes in a given combination, were SCC_low_ with few exceptions and carried fewer AMR genes on average than isolates from other clades.

**Table 1.** Number of SCC_high_ and SCC_low_ isolates encoding the specified genes and gene combinations

Gene	Gene product	RF_low_ group			RF_high_ group		
		SCC_high_	SCC_low_	Total RF_low_	SCC_high_	SCC_low_	Total RF_high_
*tfr*	TetR/AcrR family transcriptional regulator	*14*	*33*	47	*15*	*1*	16
*TRAP*	Signal transduction protein TRAP	*15*	*42*	57	*28*	*5*	33
*garR*	2-hydroxy-3-oxopropionate reductase	*15*	*42*	57	*28*	*5*	33
*T7SS*	Type VII secretion proteins	*13*	*28*	41	*7*	*0*	7
*lac972*	Lactococcin 972 family bacteriocin	*15*	*42*	57	*24*	*6*	30
*lpl*	Lysophospholipase	*14*	*33*	47	*0*	*0*	0
*azoR*	FMN-dependent NADH:quinone oxidoreductase	*15*	*42*	57	*20*	*5*	25
*fmo*	FAD-dependent monooxygenase	*14*	*33*	47	*1*	*0*	1
*dinB*	Damage-inducible protein DinB	*15*	*40*	55	*18*	*4*	22
*t7lxg*	T7SS effector LXG polymorphic toxin	*14*	*30*	44	*8*	*0*	8
*ahp*	Alkylhydroperoxidase	*15*	*42*	57	*22*	*15*	37
agrD*	Cyclic lactone autoinducer peptide	*15*	*42*	57	*30*	*6*	36
**Gene combinations**							
*tfr*, *TRAP*, *garR*, *T7SS*		*12*	*28*	0	*0*	*0*	0
*t7lxg*, *lac972*, *tfr*, *azoR*, *T7SS*		*12*	*27*	39	*0*	*0*	0
*ahp*, *lac972*, *dinB*, *t7lxg*		*14*	*30*	44	*2*	*0*	2
*t7lxg*, *lpl*		*13*	*29*	42	*0*	*0*	0
*fmo*, *lac972*, *dinB*		*14*	*33*	47	*0*	*0*	0
agrD*, *t7lxg*		*14*	*30*	44	*8*	*0*	8

* Mentioned in text, not identified as an important predictor of SCC or RF group.

## Discussion

Somatic cell count in milk samples is widely used as an indicator for IMI in dairy cows and serves as a general measure of udder health. Factors such as days in milk, antibiotic treatment history, housing conditions and previous infections can elevate SCC, though infection with mastitis pathogens such as *S. aureus* and *Streptococcus* species is a primary reason for increased inflammation [66,68]. SCC has been used as a metric to distinguish minor pathogenic bacteria causing IMI, including NAS species that are more likely to be mastitis pathogens [13]. Elevated SCC does not always reflect inflammation caused by an isolated bacterial strain, however. In some cases, the primary pathogen may not be detected if it is no longer shed in milk, was eliminated before or during sampling procedures or is non-culturable [69]. Despite these limitations, SCC can provide a useful, albeit imperfect, starting point for exploring bacterial strains associated with reduced inflammatory responses or protective effects [70]. To identify potentially probiotic isolates, we compared the genetic profiles of *S. xylosus*, *S. pseudoxylosus* and *S. shinii* isolates from samples with varying SCC in the corresponding milk samples and aimed to uncover bacterial traits linked to lower inflammation.

We explored the potential isolate-level genetic contributors to SCC variation in *S. xylosus* by dividing our isolates by SCC level (SCC_high_ and SCC_low_), using the standard threshold for subclinical mastitis [1]. We then employed multiple analyses in our approach; we built a phylogenetic tree to identify isolate groupings based on lineage, while MDS helped visualize similarities in virulence factor, bacteriocin and AMR gene content. To better distinguish isolates that were more likely to influence the level of inflammation from those where SCC levels may have been influenced by external, host or unrelated factors, we used RF classification to identify the genetic features most predictive of SCC category and then grouped isolates based on these predictors using RF clustering. This approach enabled us to focus on isolates with genomic profiles consistently associated with either high or low SCC rather than relying on SCC level as a strict indicator of NAS-triggered inflammation. Although SCC levels associated with our isolates may still partly reflect host or environmental influences, the resulting RF_high_ and RF_low_ groups offered a more nuanced interpretation for the selection of important genes and subsequent probiotic candidate identification. Finally, we used association rule learning to detect combinations of genes that were overrepresented in isolates from SCC_low_ samples with genomic profiles also associated with RF_low_.

Across all isolates, we observed a positive correlation between SCC and the number of AMR genes. We also found that the number of AMR genes was positively correlated with lantibiotic gene presence. We did not find a significant correlation between SCC level and the number of virulence factors or bacteriocins, however. This suggests that the mere presence of virulence factors or bacteriocins as a group does not directly predict the pathogenicity or protective capacity of an isolate. This is consistent with previous studies, which have not found an association between the abundance of VFs and SCC [71,74]. Rather, specific groups of virulence factors produced by pathogenic bacteria are linked to increased SCC [75], though our sample size limited our ability to examine any association between specific categories of virulence factors and SCC in greater detail. Surprisingly, we observed a negative correlation between the number of virulence factors and lantibiotic genes, and we found that the most common virulence factors were type VII secretion system genes, which have been shown to mediate interbacterial competition in *Firmicutes* and have high inter- and intra-species variation [76]. This inverse relationship could suggest an evolutionary trade-off between broad-spectrum antimicrobial production (e.g. lantibiotics) for more specific niche competition (e.g. T7SS LXG toxins, *t7lxg*). Further research into these correlations, including investigation of historical antimicrobial exposure and the possible co-selection of resistance and competitive traits, is needed.

Regardless, antimicrobial-resistant strains are not an appropriate choice for probiotic development due to the potential transfer of AMR genes to pathogenic bacteria. The correlation between lantibiotics and AMR genes also emphasizes that finding an effective probiotic is more nuanced than simply identifying bacteriocin-encoding strains. To account for this, we identified gene combinations that were disproportionately present in RF_low_ isolates. Although many of these genes were individually found in both SCC_high_ and SCC_low_ isolates, combinations of two or more were carried primarily by isolates in the RF_low_ group. These findings underscore the importance of examining multiple gene associations over individual gene presence and provide a basis for identifying probiotic potential using multigene (genomic) signatures. Further investigation of these gene combinations may yield valuable insights into traits important for probiotic activity, such as the co-occurrence of genes for niche competition and surviving host immune responses. For example, we found that the niche competition genes *lac972* and *t7lxg* often co-occurred in RF_low_ isolates alongside the oxidative stress resistance gene, *ahp*, and genes coding for lipoproteins, which sometimes modulate immune responses [77,78]. This combination of genes in a probiotic could contribute either to beneficial, localized immune stimulation or to immune suppression and evasion. Further work examining how these genes interact (e.g. through synergistic effects and changes in expression) may also help explain the observed variation in activity between strains encoding similar antimicrobial gene clusters [24,27]. Moreover, because some of the identified combinations include genes of unknown function, they offer a promising starting point for characterizing previously unannotated genes.

While our findings are based on statistical associations that have yet to be experimentally validated, bioinformatic methods have played an increasingly important role in characterizing probiotic bacteria [8,79, 80]. Though there are few examples of large-scale screening for novel candidates, genomic-scale metabolic models have been used to predict candidate probiotic strains for aquaculture applications [81] and for use in human therapeutics [82]. Additionally, in a metagenomic analysis of clinical mastitis infections, Park *et al.* [11] suggest that bacterial species negatively correlated with *S. aureus* may have probiotic potential, though employing metagenomics as a screening strategy requires refinement. We build on the growing collection of screening techniques by using machine learning algorithms alongside traditional genomic analyses to differentiate potentially beneficial *S. xylosus* isolates from those that may exacerbate IMI. To the best of our knowledge, this is the first study to use association rule learning and random forest clustering to identify biomarkers associated with inflammation or to use the absence of inflammation as a selection criterion for probiotic bacteria. It is also the first to apply machine learning and bioinformatic methods specifically for selecting novel probiotic NAS in mastitis research. Our methodology also offers broader potential applications for distinguishing more virulent strains among pathogenic species and identifying novel inflammation-related genes.

In conclusion, we found that isolates classified as SCC_low_/RF_low_ that carry combinations of the genes we have identified, particularly those from phylogenetic Clades A1, B and D1, warrant further investigation as prospective probiotic candidates. While our findings highlight promising targets, these associations remain exploratory, and both the probiotic potential of the isolates and the functional relevance of the identified gene combinations require experimental validation. Although validation was beyond the scope of this study, the antagonistic activity of these isolates against *S. aureus* is currently being assessed using assays previously developed in the Ronholm lab [25]. These gene combinations may also prove useful for detecting protective strains among other NAS species, and additional gene combinations relevant to udder health may be discovered through similar analytical approaches. This study was limited by the available dataset; to improve the resolution of associations between bacterial genes, host response and protective potential, future studies would benefit from larger sample sizes with more isolates from non-*S. xylosus* species, samples collected across multiple timepoints and the inclusion of metadata such as antimicrobial treatment history and prior infection status.

## Supplementary material

10.1099/mgen.0.001708 Uncited Supplementary Material 1.

## Abbreviations

AIPs: autoinducing peptides
AMR: antimicrobial resistance
ANI: average nucleotide identity
ARL: association rules machine learning
FP: frequent pattern growth
IMI: intramammary infection
MDS: multidimensional scaling
NAS: non-*Staphylococcus aureus* staphylococci
RF: random forest
SCC: somatic cell counts
SCC: somatic cell count
T7SS: type VII secretion system
VF: virulence factor
